# Restoring Mitochondrial Homeostasis: Therapeutic Strategies for Metabolic Dysfunction-Associated Fatty Liver Disease

**DOI:** 10.3390/ijms27062599

**Published:** 2026-03-12

**Authors:** José S. Morgado, Ivo F. Machado, Anabela P. Rolo, Carlos M. Palmeira

**Affiliations:** 1 Department of Life Sciences, Faculty of Sciences and Technology, University of Coimbra, 3000-456 Coimbra, Portugal; jose.morgado.2403@gmail.com (J.S.M.); anpiro@ci.uc.pt (A.P.R.); 2 CNC-UC—Center for Neuroscience and Cell Biology, University of Coimbra, 3004-504 Coimbra, Portugal; 3 CiBB—Center for Innovative Biomedicine and Biotechnology, University of Coimbra, 3004-504 Coimbra, Portugal; 4 Doctoral Program in Experimental Biology and Biomedicine (PDBEB), Institute of Interdisciplinary Research, University of Coimbra, 3004-504 Coimbra, Portugal

**Keywords:** MAFLD, mitochondria, biogenesis, mitophagy, dynamics

## Abstract

Metabolic dysfunction-associated fatty liver disease (MAFLD) has become the most prevalent chronic liver disorder worldwide, driven by metabolic dysfunction, excessive lipid accumulation, and progressive hepatocellular injury. A growing body of evidence identifies mitochondrial impairment as a central contributor to MAFLD pathogenesis and disease progression. Reduced oxidative capacity, elevated reactive oxygen species, and accumulation of dysfunctional mitochondria collectively exacerbate steatosis, inflammation, and metabolic inflexibility. In recent years, therapeutic strategies aimed at restoring mitochondrial homeostasis have gained considerable attention, with particular focus on agents capable of inducing mitochondrial biogenesis through pathways involving PGC-1α, AMPK, SIRT1, and mTOR. This review synthesizes current knowledge on mitochondrial dysfunction in MAFLD and highlights emerging compounds that ameliorate disease phenotypes by enhancing mitochondrial biogenesis. By examining their mechanisms of action and preclinical efficacy, we underscore the therapeutic potential of targeting mitochondrial quality-control pathways, mainly mitochondrial biogenesis, as a promising avenue for mitigating MAFLD progression.

## 1. Introduction

Metabolic diseases are illnesses that tamper with the body’s normal metabolic reactions, usually via a congenital lack of a crucial enzyme involved in the biosynthesis or degradation of biomolecules. They can also result from dysfunctions in signaling molecules or hormones, whose dysregulation disrupts the body’s natural ability to regulate metabolism. Among these metabolic illnesses, the most prevalent liver condition is Metabolic dysfunction-associated fatty liver disease (MAFLD), which has affected an estimated 1.2 billion people worldwide as of 2019 [1]. It affects men more commonly than women (males—40%; females—26%) [2]. This disease is defined by a hepatic lipid content above 5% and is characterized by an increase in hepatocyte lipid storage (steatosis). For accurate diagnosis there needs to be confirmation of an increase in triglyceride storage in the liver by imaging or histology, as well as exclusion of major alcohol consumption (over 40 g daily) [3]. Steatogenic medication consumption, as well as preexisting liver conditions, must likewise be ruled out, as is the case for methotrexate intoxication, Wilson’s disease, hemochromatosis, tyrosinemia, viral hepatitis and pregnancy [3]. Simple steatosis can progress to steatohepatitis (NASH), cirrhosis and even hepatocellular carcinoma [4]. Steatosis is defined as fatty liver with no hepatocellular damage or hepatocyte ballooning, while steatohepatitis shows both of these signs with or without fibrosis [5,6]. The main risk factors for the development of this pathology includes an age of 50 years or older, type 2 diabetes mellitus, obesity and insulin resistance [7]. MAFLD pathogenesis is best explained, as of today, by the multiple-hit hypothesis. Stemming from the two-hit hypothesis [8], this theory proposes that hepatic steatosis occurs due to a series of parallel insults, among which insulin resistance is a key player leading to an increase in lipogenesis and subsequent accumulation of free fatty acids in the liver [9]. This accumulation then leads to inflammation which in turn leaves the liver susceptible to further insults [9,10]. A growing body of evidence has emerged demonstrating that mitochondrial dysfunction is an important player in MAFLD pathogenesis. Reduced fatty acid oxidation as well as a reduction in mitochondrial markers for mitochondrial biogenesis, mitochondrial dynamics and mitophagy have been observed in human hepatocytes obtained via biopsy [11]. However, some studies reveal that an increase in fatty acid oxidation happens instead, being at least partially responsible for an increase in reactive oxygen species (ROS) which can exacerbate MAFLD due to oxidative damage [12]. This increase is explained by an increase in compensatory beta-oxidation in peroxisomes, to alleviate mitochondrial burden. Additionally, Peroxisome proliferator-activated receptor coactivator 1-⍺ (PGC1⍺) protein levels were shown to be significantly decreased in fatty liver mouse models, alongside genes regulated by PGC1⍺ such as mitochondrial transcription factor A (*TFAM*) and nuclear respiratory factor 1 (*NRF-1*) [13]. This leads to an accumulation of a dysfunctional mitochondrial pool that aggravates the MAFLD phenotype. Currently, there are only two FDA-approved treatments for this malady (resmetirom and semaglutide), highlighting the need for the development of new therapies. Treatments targeting the mitochondria have shown great promise in recent years, being able to ameliorate disease phenotypes by rescuing mitochondrial function [14]. Particularly, the induction of mitochondrial biogenesis has been shown to be one of the main mechanisms of improving mitochondrial function for several of these treatments, showing that it is a viable therapeutic strategy to combat MAFLD. In this review we will shine light on novel MAFLD therapies that help improve mitochondrial function, with a focus on mitochondrial biogenesis inducers, as well as elucidating the mechanisms behind each compound’s induction.

## 2. MAFLD Pathogenesis

### 2.1. Epidemiology and Histological Features

**Figure 1:**
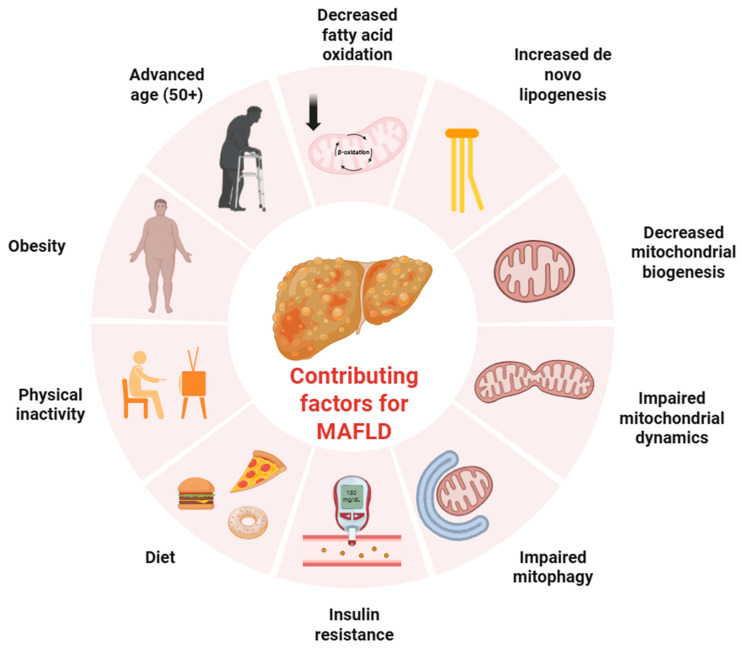
Schematic representation of factors contributing to the progression of MAFLD. Unhealthy eating and physical activity habits, alongside several of their consequences such as obesity and insulin resistance, are major risk factors in the development of MAFLD. Defective lipid metabolism, including increased de novo lipogenesis and decreased fatty acid oxidation leads to lipid accumulation in the liver. Impaired mitochondrial parameters such as impaired dynamics and a reduction in mitophagy and biogenesis result in a fragilized mitochondrial network that worsens disease progression.

MAFLD has been dubbed the hepatic manifestation of metabolic syndrome [10]. It is by far the most prevalent liver disease worldwide, having seen its global prevalence increase from 26% (from studies conducted in 2005 or prior) to 38% (2016 and beyond) [2]. A study analyzing the global burden of MAFLD conducted between 1990 and 2019 projects a steady and significant climb in cases for the near future, deeming this disease a great concern for public health [15]. MAFLD has been established as an independent risk factor for several different types of extrahepatic diseases that contribute significantly to disease severity and morbidity (Figure 1). MAFLD raises the risk of development of cardiovascular disease, displaying altered cardiac structure and an increased risk of developing ischemic heart disease [16,17]. Risk of developing chronic kidney disease (CDK) is also well documented, with the co-incidence of CKD and MAFLD ranging from 20 to 55% compared to 5–30% in patients without MAFLD [18,19,20]. Additionally, diabetic populations seem to suffer more from MAFLD than non-diabetics with 55–70% of patients with T2DM presenting with MAFLD, 30–60% presenting with NASH and around 12–20% demonstrating signs of cirrhosis [15,21,22].

As previously mentioned, MAFLD is characterized by a steatotic hepatocyte content above 5% [23]. Steatosis in MAFLD is most frequently macrovesicular, meaning that affected hepatocytes display a large cytoplasmic fat droplet or several small and isolated droplets which displace the nucleus to the cell periphery [23]. MAFLD is a progressive disease, with around 20% of individuals progressing from MAFLD to NASH, and 20% of those with NASH will further progress to cirrhosis [24,25]. Distinction amongst these levels of progression is done via histological analysis of liver biopsies, in which a scoring system is applied to evaluate the severity of disease [23,26]. Distinction between MAFLD and NASH comes about when the usual MAFLD histology (macrovesicular steatosis) also presents significant damage to hepatocytes in the form of hepatocellular ballooning and inflammation [26]. Although NASH can present with significant levels of fibrosis, it does not always do so, making its presence not a necessity for diagnostic purposes [23,26]. NASH shows a histological distribution that seems to favor acinar zone 3 of the liver, with hepatic stellate cell and Kupffer cell activation present in this zone [26,27]. As the disease worsens, the likelihood of fibrosis appearing rises, with its measure serving as the main indicator of long-term outcomes in patients with MAFLD [26,28].

### 2.2. Lipid Transport

The main hallmark of MAFLD pathogenesis is the accumulation of fatty acids in the liver. A total of 60% of hepatic fat content is derived from free fatty acids originating from adipose tissue lipolysis, while lipids derived from de novo lipogenesis (DNL) and dietary consumption make up 30% and 10%, respectively [29,30]. Since fatty acids are lipophilic, they circulate in the bloodstream attached to carrier proteins such as albumin. These free fatty acids diffuse through the plasma membrane unassisted or via caveolin-mediated endocytosis. Additionally, they can be transported by fatty acid translocases (FATs) such as CD36 and via fatty acid transport proteins (FATPs) [31]. In regard to FATPs, the liver mainly expresses the following two: FATP2 and FATP5 [32]. Changes in FATP5 have been associated with the exacerbation of the metabolic syndrome phenotype and the severity of MAFLD [30]. A study investigating polymorphisms in the FATP5 promoter discovered that MAFLD patients carrying the A-allele had increased alanine transaminase (ALT) activity [33]. Additionally, this study proposed that the impact of BMI in MAFLD was dependent on the FATP5 polymorphism. Recent articles show that inhibiting hepatic fatty acid uptake by silencing FATP5 via RNA interference, it is possible to reverse diet-induced MAFLD while also improving hypoglycemia [34]. This suggests that diet-induced MAFLD is regulated by continuous fatty acid uptake via FATP5 [34]. As for FATP2, there is a correlation between its expression and MAFLD progression [35]. FATP2, alongside CD36, are upregulated in NASH, leading to an increase in long chain fatty acid uptake [35]. Knocking down FATP2 via RNA interference, similarly to FATP5, has a protective effect against MAFLD in mice [36].

### 2.3. De Novo Lipogenesis

DNL is a major contributor to the hepatic free fatty acid pool with patients suffering from MAFLD showing a significant increase in DNL [37]. Lipid biosynthesis starts with the carboxylation of acetyl-CoA to malonyl-CoA by Acetyl-CoA Carboxylase, the rate limiting enzyme of fatty acid synthesis [38]. This malonyl-CoA will serve as an acetyl group donor, essential for the elongation of the carbon chain. Fatty acid elongation occurs by a series of reactions catalyzed by several enzymes comprising a larger enzymatic complex called fatty acid synthase (FAS). These reactions occur cyclically in sets of four, in which the saturated acyl group generated by these reactions serves as the substrate for further elongation. Before these series of reactions occurs, an acetyl group from acetyl-CoA must be transferred to the Acyl-carrier Protein (ACP) by malonyl/acetyl-CoA–ACP transferase, after which it is transferred to the β-ketoacyl-ACP synthase (KS) domain of FAS. Only after this addition is complete can the four-step reaction cycle start, via addition of malonyl groups to ACP and subsequent transfer to the already existing acetyl moieties bound to the KS domain. With each cycle that passes, the carbon chain grows longer by two carbons. After seven cycles, the palmitoyl group is hydrolyzed from the ACP, generating free palmitate. It is from palmitate that other longer saturated fatty acids are synthesized, not via FAS activity, but instead through the action of fatty acid elongation systems. Unsaturated fatty acids are synthesized by a series of desaturases, utilizing both endogenously synthesized lipids such as palmitate and stearate (to yield palmitoleate and oleate) as well as already oxidized and longer fatty acids obtained in the diet [39,40]. Although fatty acids synthesized from scratch, such as palmitate, stearate, palmitoleate and oleate, are used in triglyceride synthesis, they only make up about 15–25% of the fatty acids present in them, with dietary fatty acids showing a bigger prevalence in the hepatic triglyceride pool [29]. To form triglycerides, available fatty acids must bind to glycerol. L-glycerol-3 phosphate is acetylated in both its free hydroxyl groups by two fatty acyl-CoA molecules, forming diacylglycerol 3-phosphate or phosphatidic acid. The enzyme lipin then hydrolyzes it, removing the phosphate group and forming 1,2-diacylglycerol, which will be esterified by a third fatty acyl-CoA, by diacylglycerol acetyltransferase (DGAT), to yield a triglyceride. These processes are thoroughly regulated by transcription factors such as sterol regulatory element binding protein 1C (SREBP1C) and carbohydrate response element binding protein (ChREBP) as well as by hormones such as insulin. SREBP1C activates the expression of several proteins involved in hepatic DNL such as ACC, FAS, stearoyl-CoA desaturase-1 and Glycerol-3-phosphate acetyltransferase [41,42]. SREBP1C has been demonstrated to play a role in altered DNL in MAFLD, having its gene expression upregulated in individuals carrying this pathology [43]. Furthermore, overexpression of this protein in the liver leads to increased hepatic lipid accumulation and DNL, as well as alterations in liver morphology and hepatic lipid profile [44]. Another transcription factor, liver X receptor α (LXRα), acts through SREBP1C to regulate DNL [45]. Its expression has also been found to be elevated in MAFLD patients [46,47]. As for ChREBP, its liver-specific inhibition improved hepatic steatosis in leptin-deficient ob/ob mice, with these mice showing improved insulin resistance and glucose tolerance [48]. However, C57BL/6J mice overexpressing ChREBP on a high-fat diet also displayed increased insulin sensitivity and glucose tolerance despite also developing hepatic steatosis, which could mean that insulin resistance is not directly caused by ChREBP-induced fatty liver [49].

### 2.4. Lipotoxicity

In MAFLD, there is an elevation of circulating free fatty acids, as well as a decrease in fatty acid oxidation [50,51]. This shift leads to an increase in total lipid content in the body. Lipids are very heterogeneous in function, having different roles such as energy storage and cellular membrane formation, as well as serving as synthetic precursors for signaling molecules [52]. Despite their physiological roles, certain lipids can do more harm than good when it comes to MAFLD pathogenesis, especially when present in chronically elevated concentrations. Both in vitro and in vivo studies demonstrated a higher toxicity of saturated fatty acids such as palmitate and stearate when compared to unsaturated ones such as palmitoleate and oleate [53,54]. Saturated fatty acids induced lipoapoptosis and decreased autophagy, whereas unsaturated fatty acids did not [53]. Additionally, saturated fatty acids increase insulin resistance, lower lipolysis and increase hepatic triglyceride content, while their unsaturated counterparts show opposite effects [54]. A proposed mechanism to explain the cytoprotective effects of unsaturated fatty acids suggests that they promote saturated fatty acid clearance through enhancement of their esterification and lipid droplet incorporation, as well as by increasing fatty acid oxidation [55].

Palmitate has been shown to lead to damage in biomolecules such as lipids, proteins and even DNA, while also activating caspase-dependent apoptosis in HepG2 cells [56]. This was paired with an increase in reactive oxygen species (ROS) and an overexpression of inflammatory markers such as Interleukine-6 (IL-6) and Tumor necrosis factor- α (TNF-α). Cells treated with palmitate also demonstrated increased translocation of Nuclear factor kappa-light-chain-enhancer of activated B cells (NF-kB) from the cytosol, indicating an increase in both inflammation and redox signaling [56]. Furthermore, a study showed that palmitate toxicity is associated with DGAT1 downregulation and led to a reduction in Peroxisome proliferator-activated receptor alpha (PPARα) levels in human hepatocellular carcinoma cell lines [57]. DGAT1 downregulation prevented palmitate detoxification via esterification into triglycerides, leading to an exacerbation of lipotoxicity. Additionally, PPARα agonists were able to rescue some of these defects by restoring fatty acid oxidation capacity and DGAT1 expression.

Stearate exerts its lipotoxic mechanisms by mechanisms similar to palmitate’s possibly due to their similar chemical structure. In circulating angiogenic cells, stearate activated effector caspases and increased gene expression of the following pro-inflammatory markers: *IL-6*, *IL-8*, *IL-1β*, Monocyte chemoattractant protein-1 (*MCP-1*) and *TNFα* [58]. Stearate-induced lipotoxicity has also been observed in pancreatic β-cells, where it upregulates miR-34a-5p and subsequently inhibits anti-apoptotic proteins BCL-2 and BCL-W [59].

Fatty acids are not the only lipids that exert lipotoxicity in MAFLD. Excess cholesterol accumulation in hepatocytes also contributes to disease severity, dependent on its ratio to high-density lipoprotein cholesterol [60]. One study showed that cholesterol can form crystals in hepatocyte lipid droplets in mice fed a high-fat, high-cholesterol diet [61]. Crystal deposition activated Kupffer cells, inducing their degradation of the crystals and conversion into lipid-laden foam cells similar to those found in atherosclerosis [62]. Additionally, the study demonstrated that exposure of HepG2 cells to LDL cholesterol crystal formation has activated cocultured THP1 macrophages, with subsequent upregulation of TNF-α, NLRP3 and IL-1β transcripts [61]. Several studies have linked cholesterol to mitochondrial dysfunction in several different cell types [63,64,65]. Besides alteration of mitochondrial membrane fluidity, excess cholesterol increases mitochondrial ROS-mediated damage by not only increasing its production, but also by lowering mitochondrial glutathione levels [66].

### 2.5. Phosphatidylethanolamines

Phosphatidylethanolamine (PE) is one of the most abundant phospholipids in eukaryotic cells, comprising roughly 15–25% of total cellular phospholipids [67]. Its biophysical properties, particularly its cone-shaped geometry, promote negative curvature [68]. This curvature is essential for maintaining the mitochondrial cristae structure, which in turn determines the efficiency of oxidative phosphorylation [68].

Mitochondrial PE is synthesized primarily by phosphatidylserine decarboxylase (PSD/PISD), an inner mitochondrial membrane enzyme that converts phosphatidylserine (PS) to PE. The importance of this pathway becomes evident when it is perturbed. A study demonstrated that even a slight reduction in mitochondrial PE (20–30%) is sufficient to impair mitochondrial bioenergetics [69]. In both acute PISD knockdown and chronically PE-deficient PSB-2 cells, the authors observed reduced respiratory capacity, decreased ATP production, and diminished activities of complexes I and IV. Blue native PAGE revealed defective assembly of respiratory supercomplexes, accompanied by reduced steady-state levels of Complex I and Complex IV proteins [69]. These defects occurred despite normal or elevated levels of mitochondrial fusion proteins Mitofusin 1 (MFN1), MFN2 and optic atrophy 1 (OPA1), indicating that the extensive mitochondrial fragmentation and aberrant ultrastructure observed in PE-deficient cells arise from intrinsic membrane defects rather than from canonical fission-fusion pathways.

Beyond its structural and bioenergetic roles, mitochondrial PE is intimately tied to mitochondrial protein import and quality control. Sam et al. showed that when PE metabolism is severely impaired, mutant forms of Psd1 that fail to undergo autocatalytic processing accumulate in the endoplasmic reticulum [70]. This leads them to become N-glycosylated, then ubiquitinated and finally degraded. This mislocalization occurs only under conditions of profound PE deficiency and reflects an adaptive stress response that monitors mitochondrial precursor proteins during import [70]. The authors conclude that impaired PE metabolism activates a reversible ER-assisted surveillance mechanism capable of identifying and resolving nonfunctional mitochondrial precursors.

The spatial regulation of PE synthesis is even more complex than previously thought. Findings from one study revealed that Psd1 is not exclusively mitochondrial but is also targeted to the ER, where it generates a distinct pool of PE with non-redundant cellular functions [71]. PE synthesized in mitochondria is essential for maintaining mitochondrial morphology and respiratory function, whereas ER-localized Psd1 supports global cellular PE homeostasis. The relative distribution of Psd1 between mitochondria and ER shifts depending on metabolic conditions, thus demonstrating the dynamic regulation of PE synthesis in response to cellular demand.

Recent investigation has demonstrated that PE dysregulation plays an important role in MAFLD pathogenesis. Shama et al. provided direct evidence that specific PE species accumulate in the serum of obese individuals with progressive NAFLD/MAFLD [72]. PE (34:1), PE (36:2), and PE (34:2) levels increased linearly with disease progression. Importantly, these PE species exerted pathogenic effects in vitro. In HepG2 hepatocytes, treatment with PE (34:1) or PE (36:2) reduced mitochondrial mass and membrane potential and increased mitochondrial superoxide production [72]. These mitochondrial defects were accompanied by transcriptional changes consistent with disrupted lipid homeostasis: upregulation of FASN, SREBP, DGAT1, CPT, and PPARα, and activation of apoptotic pathways (increased BCL2-associated X protein (BAX) and cytochrome c, as well as decreased BCL2) [72]. The authors also observed increased LC3 expression and reduced mTOR expression, indicating activation of autophagy and mitophagy-related signaling in response to PE-induced mitochondrial stress [72].

In hepatic stellate cells, the same PE species promoted migration and activation, with increased expression of inflammatory proteins such as TGF-β, TNF-α, and IL-6 [72]. These findings demonstrate that elevated PE species promote mitochondrial dysfunction, oxidative stress, impaired mitophagy signaling, and fibrogenic activation, all of which are key pathological features of MAFLD progression.

Additional support for the pathogenic consequences of PE dysregulation comes from hepatocellular carcinoma models. A recent study showed that silencing PISD in HEPA1–6 liver cancer cells reduces impairs mitochondrial respiration, decreases complex I and IV levels, increases mitochondrial superoxide production, and enhances mitochondrial fission and mitophagy [73]. These mitochondrial defects were accompanied by reduced DNA synthesis, decreased proliferation, and diminished mTOR signaling.

Finally, alterations in PE metabolism outside mitochondria also influence hepatic lipid storage and energy homeostasis. It was recently demonstrated that expression of Ethanolamine phosphate phospholyase, an enzyme that degrades phosphoethanolamine (the substrate for the Kennedy pathway) leads to detrimental effects in Huh7 hepatoma cells [74], among which are increased neutral lipid storage, decreased ATP production, and reduced oxygen consumption. These metabolic disturbances were accompanied by reduced proliferation and increased expression of tumor suppressor genes (*CDKN1A*, *BBC3*, *BAX*, and *BRCA1*) [74]. The increased PC:PE ratio observed in this model mirrors changes associated with MAFLD and impaired mitochondrial function.

## 3. Mitochondrial Dysfunction in MAFLD

Mitochondria are complex organelles and often referred to as “the powerhouse of the cell”. However, their role in cellular function is much more diverse, acting as the hub of cellular metabolism and as regulators of ATP synthesis, redox balance, programmed cell death and cell signaling [75]. Mitochondria’s most well-known function is ATP production via the electron transport chain (ETC). The ETC is made up of four membrane-bound proteins that transport electrons while building an electrochemical gradient from the reactions that occur. This occurs via coupling of proton pumping into the intermembrane space, which will then be shuttled back into the mitochondrial matrix. This shuttling, when assisted by ATP synthase, leads to the formation of ATP and electron transport to ½ O_2_ yielding H_2_O. Mitochondria are the primary site of β-oxidation of fatty acids, converting long-chain acyl-CoAs into acetyl-CoA while generating reducing equivalents (NADH, FADH2) that feed the ETC. Besides ATP synthesis the ETC is also the main site of ROS production. Electron leaks from the respiratory complexes, mainly I and III, lead to the formation of superoxide which is rapidly converted to hydrogen peroxide by mitochondrial superoxide dismutase (mSOD). While excessive mitochondrial ROS lead to oxidative cellular damage, sublethal ROS levels function as signaling molecules. Through reversible oxidation of cysteine and methionine residues in redox-sensitive proteins, ROS modify the conformation and activity of several phosphatases, kinases, proteases and transcription factors [76]. To execute these functions effectively, the mitochondrial pool must remain healthy. The health of the mitochondrial pool is maintained by several quality-control mechanisms, with these being mitochondrial fusion and fission, mitophagy and biogenesis. These processes work together in order to adapt mitochondrial morphology to ever-changing cellular needs proving themselves to be crucial in maintaining physiological homeostasis.

**Figure 2:**
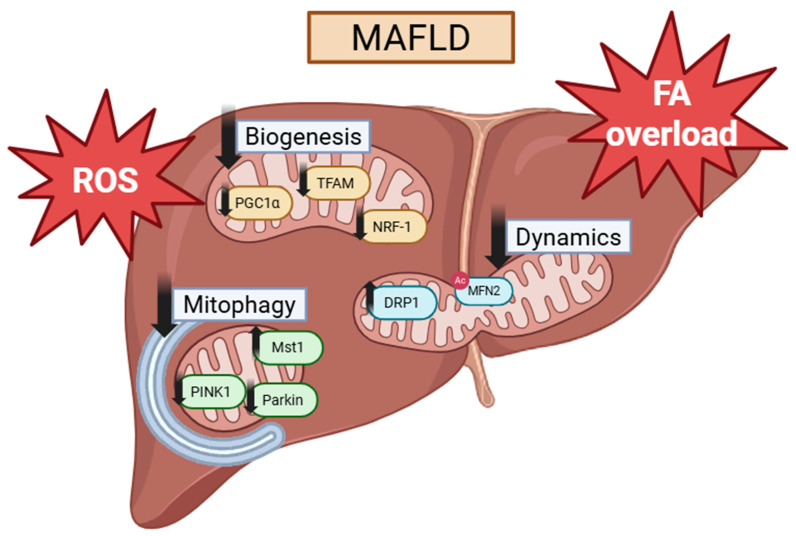
Schematic representation of mitochondrial dysfunction in MAFLD. Fatty acid overload alongside ROS stress strain the mitochondrial network, leading to defective biogenesis, dynamics and mitophagy. In the case of mitochondrial biogenesis, mitochondria show a decrease in important markers such as PGC1α, TFAM and NRF-1, indicating impaired signaling in mitochondrial biogenesis-related pathways. An increase in DRP1 accompanied by MFN2 acetylation worsens mitochondrial dynamics, leading to a fragmentation of the mitochondrial network. Impaired PINK1-PARKIN signaling alongside an increase in Mst1 levels results in impaired mitophagy. MAFLD: Metabolic dysfunction-associated fatty liver disease; ROS: reactive oxygen species; FA: fatty acid; PGC1α: Peroxisome proliferator-activated receptor gamma coactivator 1 alpha; TFAM: mitochondrial transcription factor A; NRF-1: nuclear respiratory factor 1; DRP1: dynamin related protein 1; MFN2: Mitofusin 2; PINK1: PTEN-induced kinase 1; Mst1: Macrophage stimulating 1.

In MAFLD, all of these mitochondrial processes have been demonstrated to be impaired in one way shape or form, resulting in impaired energy metabolism, increased ROS production, defective mitophagy and impaired biogenesis. These changes collaboratively worsen MAFLD phenotype, proving to be essential in disease pathology (Figure 2).

### 3.1. Mitochondrial Biogenesis

Since mitochondria are so heavily impacted in MAFLD, it is imperative to understand the mechanisms behind mitochondrial quality control, namely, mitochondrial biogenesis, mitochondrial dynamics and mitophagy. Jornayvaz et al. defines mitochondrial biogenesis as the growth and division of pre-existing mitochondria [77]. This process is essential for the maintenance of a healthy mitochondrial pool, as it is responsible for the renewal of mitochondria, leading to a better response to the cell’s metabolic needs. PGC1α, usually referred to as the master regulator of mitochondrial biogenesis, is essential in this process. It upregulates NRF-1 and NRF-2, which are responsible for the activation of genes such as *TFAM* [78]. TFAM binds sequence specifically to mitochondrial promoter regions to recruit RNA polymerase and transcription factor B2, being responsible for the transcription of 13 protein-coding genes. It also coats mtDNA non-specifically to pack it into compact nucleoids that stabilize and protect the genome from damage. Mitochondrial biogenesis can be induced in several different ways. NRF-1 and NRF-2 are also responsible for the expression of other nuclear genes important for mitochondrial function, such as genes encoding for mitochondrial respiratory complexes or heme biosynthesis enzymes [79]. There are several ways by which mitochondrial biogenesis can be induced. ATP depletion by complex I inhibitors such as metformin or by exercise leads to an increase in the AMP/ATP ratio which activates Adenosine monophosphate-activated protein kinase (AMPK) [77]. AMPK phosphorylates PGC1α directly, thus increasing mitochondrial biogenesis. AMPK also activates sirtuin 1 (SIRT1), which in turn deacetylates PGC1α, driving up oxidative metabolism and mitochondrial content. mTOR is also a known inducer of PGC1α. mTOR directly influences PGC-1α to induce mitochondrial biogenesis and oxidative metabolism. In skeletal muscle, mTOR forms a complex with the transcription factor YY1 and PGC1α, binding promoters of mitochondrial oxidative genes. Inhibition of mTORC1 by rapamycin disrupts YY1’s interaction with PGC1α, leading to decreased mitochondrial DNA content and oxygen consumption [80]. mTOR is also capable of interacting with mitochondrial proteins to influence respiratory function without relying on transcription [81].

Mitochondrial biogenesis is frequently impaired in MAFLD. A study conducted on C57BL/6J mice which were fed a choline-deficient, ethionine-supplemented diet demonstrated that PGC1α mRNA and protein levels are significantly decreased in MAFLD [13]. This was accompanied by a decrease in PGC1α-regulated proteins such as NRF-1 and TFAM by 30%. A decrease in mitochondrial electron transport chain proteins was also observed alongside elevated levels of superoxide after 14 days on the diet [13]. PGC1α also acts through SIRT3, an important modulator of mitochondrial biogenesis [82]. Knock-downs of SIRT3 in C2C12 myotubes decreased the stimulatory effects of PCG1α on mitochondrial biogenesis [83]. Although SIRT3 expression does not seem to be affected in MAFLD, its activity is markedly decreased [84]. This leads to a hyperacetylation of several mitochondrial proteins such as ETC proteins, enzymes involved in gluconeogenesis, and proteins involved in homocysteine metabolism, culminating in a disruption of lipid metabolism [84]. Furthermore, a loss of mitochondrial biogenesis leads to the acceleration of beige to white adipose tissue, thus increasing adipose tissue quantity [85]. This increase can contribute to insulin resistance, driving MAFLD pathogenesis forward. Conversely, there exists evidence demonstrating an increase in mitochondrial content in the early stages of MAFLD. Studies conducted on human MAFLD patients showed a 3.7- and 3.9-fold increase in mtDNA content when compared to healthy controls [86,87]. However, there is no consensus on whether this increase in mitochondrial quantity stems from an initial increase in biogenesis or from impaired mitophagy. Low-level lipid accumulation present in the early stages of MAFLD upregulates PPARα expression, as well as AMPK phosphorylation [88]. However, these changes were not observed in traditional fat accumulation models, suggesting a mere transient upregulation of the mitochondrial biogenesis pathway in early MAFLD.

### 3.2. Mitochondrial Dynamics

Complementing mitochondrial biogenesis, mitochondrial fusion and fission, collectively known as mitochondrial dynamics, are responsible for the regulation of the mitochondrial network. Mitochondrial dynamics is essential to repair damaged mitochondrial components and isolate damaged regions for removal. These processes are extensively mediated by a series of proteins. MFN1 and MFN2 are essential proteins for outer mitochondrial membrane (OMM) fusion [89]. MFN1 and MFN2 are OMM GTPases that initiate fusion by joining adjacent mitochondria via interactions of their heptad-repeat 2 (HR2) coiled-coil domains, forming homo-dimeric (MFN1–MFN1 or MFN2–MFN2) and hetero-dimeric (MFN1–MFN2) complexes on opposing membranes [89]. GTP binding and hydrolysis drive conformational changes that draw membranes into close apposition and promote lipid bilayer merging. MFN1 exhibits higher GTPase and tethering activity than MFN2 to catalyze the initial docking step. Loss of either protein results in fragmented mitochondria due to impaired outer membrane fusion [89]. For inner mitochondrial membrane (IMM) fusion, OPA1 is the mediator instead. OPA1, much like MFN1 and MFN2, is also a GTPase, although it acts on the IMM instead. On the other hand, mitochondrial fission is mediated by dynamin-related protein 1 (DRP1). It is recruited to the OMM, leading to both OMM and IMM constriction, which results in the separation of a single mitochondrion in two.

The interplay between mitochondrial fusion and fission is essential for maintaining a healthy mitochondrial network. Longer mitochondria are more efficient in ATP generation, due to their higher oxidative capacity. Additionally, damaged mitochondria can compensate for their deteriorated functions by fusing to healthier mitochondria [90]. This allows them to combine components, leading to the dilution of damaged proteins and mtDNA. This can lead to the restoration of membrane potential and recovery of respiratory efficiency. Conversely, a smaller and more fragmented mitochondrial network is usually a sign of oxidative damage. Mitochondria also tend to appear fragmented when there is a need for mitochondrial clearance via mitophagy [90]. This can happen in situations of energy stress, such as nutrient deprivation or exercise. In these situations, the increase in the AMP/ATP ratio activates AMPK, which in turn phosphorylates DRP1. This leads to an increase in mitochondrial fission, making the individual mitochondria smaller, which allows for an easier disposal of damaged mitochondria.

An increase in DRP1 levels has been correlated with increased disease severity in MAFLD. DRP1 expression was shown to be further increased with disease progression, having its levels increased from MAFLD to NASH and NASH-related fibrosis, setting DRP1 as a potential biomarker of MAFLD-/NASH-induced inflammation and disease progression [91]. Complementing these data, mitochondrial fission inhibition in mice led to beneficial effects such as decreased hepatocyte lipid accumulation and diminished oxidative stress [92]. Moreover, DRP1 knock-out mice protected them from diet-induced obesity and metabolic dysregulation [93]. This loss of DRP1 led to endoplasmic reticulum stress and activated eukaryotic translation factor 2α–activating transcription factor 4–FGF21 pathway, thereby increasing energy consumption [93]. Studies conducted in male MAFLD mice also showed that an increase in the levels of mitochondrial fusion proteins via exercise, namely MFN2, had several beneficial effects in the pathogenesis of the disease [87]. These mice showed a large increase in mitochondrial β-oxidation specifically in lipid droplet-bound mitochondria, which is a process in which MFN2 is essential [94]. MFN2 mediates mitochondria–lipid droplet membrane contact by binding to heat shock protein cognate 70 (Hsc70), thus enabling fatty acid transport from lipid droplets to mitochondria for β-oxidation [95]. In conditions of lipid overload, MFN2 is acetylated and degraded by the proteasome, ultimately leading to lipid accumulation in cardiomyocytes [95]. Despite this, a recent study conducted on transgenic mice with liver-specific knock-out of mitochondrial fission factor (Mff) showed that when a high-fat diet was fed, NASH developed [96]. Mff deficiency in mice led to impaired triglyceride secretion from the liver, as well as an increase in endoplasmic reticulum stress markers. These results clash with previous demonstrations of mitochondrial fission inhibition in MAFLD models. It has been established that in some MAFLD patients there is a presence of giant mitochondria, although whether this is a cause of MAFLD or a consequence is still largely up for debate [97]. One study reported that knocking out OPA1 resulted in the reversion of the megamitochondria phenotype, in addition to ameliorating the MAFLD/NASH phenotype [98]. Despite improvements in steatosis progression, fibrosis, inflammation and liver damage were still present to some extent, possibly indicating that mitochondrial morphological changes are not the sole contributor to MAFLD progression [98]. Additionally, a different study demonstrated that OPA1 stabilization resulted in an increase in mitochondrial respiration, but no changes in mitochondrial function nor lipid accumulation were observed [99]. Taken together, these suggest that the alteration in mitochondrial morphology, namely the formation of megamitochondria, might be an adaptive remodeling to increase mitochondrial respiration while facing metabolic stress. However, excessive or dysregulated fusion can turn dysfunctional, resulting in impaired mitochondrial quality control and contributing to the worsening of MAFLD phenotype.

### 3.3. Mitophagy

The process by which these mitochondria are selectively removed is called mitophagy. During this process, mitochondria are engulfed in autophagosomes for later lysosomal fusion. Mitophagy is activated under both basal and stress conditions [100]. Nutrient deprivation, hypoxia, membrane depolarization, or elevated ROS all trigger stabilization of the kinase PTEN-induced kinase 1 (PINK1) on the outer mitochondrial membrane, recruitment of the E3 ligase Parkin, and ubiquitination of outer-membrane proteins such as nuclear dot protein 52 (NDP52) and optineurin. In parallel, proteins such as BCL2-interacting protein 3 (BNIP3), NIP-3-like protein X (NIX) and FUN14 domain containing 1 (FUNDC1) as well as ubiquitin-binding adaptors such as sequestosome-1 (SQSTM1/p62) recruit microtubule-associated protein 1A/1B-light chain 3 (LC3) positive membranes to damaged mitochondria, ensuring their efficient clearance.

It has been demonstrated that both human-derived hepatocytes and mouse models of MAFLD show reduced PINK1-Parkin signaling as well as impaired LC3 recruitment and SQSTM1/p62-positive mitochondria accumulation [101,102]. In hepatocyte models of lipotoxicity, an overexposure to saturated fatty acids seemed to suppress PINK1-Parkin-dependent mitophagy, leading to reduced LC3-dependent autophagosome formation [102]. Conversely, pharmacological restoration of PINK-Parkin signaling in HepG2 cells had a protective effect against lipotoxicity. In diet-induced MAFLD, hepatocytes exhibit mitochondrial stasis, characterized by the accumulation of damaged mitochondria with p62 and ubiquitin bound to outer mitochondrial membrane proteins [101]. Enhancement of p62-mediated, Parkin-independent mitophagy pathway was shown to mitigate steatosis and liver injury in vivo.

A different study placed mitophagy impairment as an early defect of MAFLD, having found it to be diminished even before showing signs of inflammation or fibrosis [103]. The authors demonstrated a reduction in mitochondrial delivery to lysosomes, accompanied by accumulation of dysfunctional ROS producing mitochondria. Importantly, when Parkin was selectively knocked down in hepatocytes, these early defects were severely increased. These liver-specific Parkin knock-out mice developed steatosis quicker than control mice and showed increased lipid droplet accumulation [103]. Building on the previous observations, further evidence demonstrated that upstream regulators of mitophagy are also dysregulated in MAFLD. Zhou et al. identified Macrophage stimulating 1 (Mst1) as a negative regulator of Parkin-mediated mitophagy. Mst1 activity was shown to be elevated in both dietary and genetic models of MAFLD as well as in hepatocytes exposed to lipotoxic stress. Increased Mst1 signaling suppressed Parkin translocation to damaged mitochondria, thus resulting in reduced ubiquitination of outer-membrane proteins and ultimately impaired mitophagy. Mst1 knock-down restored Parkin-dependent mitophagy, significantly easing steatotic burden and oxidative stress.

Broader analyses of mitochondrial dysfunction in MAFLD show that compromised mitophagy and mitochondrial quality control as a whole are intimately involved in disease progression [104,105]. Reduced mitophagy promotes an increase in mitochondrial ROS production and suppressed β-oxidation, leaving hepatocytes fragile and more susceptible to lipotoxic injury [106].

Recent research has uncovered hepatocyte-specific regulatory mechanisms that directly connect defective mitochondrial clearance to the progression of NASH and the inflammatory microenvironment that promotes tumor development. Loss of the transcription factor Myc-interacting zinc-finger protein 1 (Miz1) in hepatocytes, as observed in human NASH and diet-induced mouse models, disrupts mitophagy by permitting the translocation of cytosolic peroxiredoxin 6 (PRDX6) to the OMM [107]. There, PRDX6 binds Parkin at Cys431, blocking Parkin autoubiquitination and preventing ubiquitin-dependent recruitment of the autophagy machinery [107]. This inhibition of mitophagy leads to accumulation of swollen, ROS-producing mitochondria and amplifies TNFα-driven inflammatory loops that accelerate steatohepatitis and fibrosis [107]. Importantly, this positive feedback loop helps explain why NASH becomes self-sustaining and irreversible, solidifying its progression into HCC.

In parallel, impaired mitophagy contributes to the oncogenic environment characteristic of HCC. Mitophagy-related genes such as optineurin (*OPTN*), Autophagy-related 12 (*ATG12*), *MFN1*, *SQSTM1*, Translocase of outer mitochondrial membrane 22 (*TOMM22*), and *TOMM5* are dysregulated in HCC [108]. This has shown a correlation with poor prognosis and reduced antitumor immune competence [108].

Together, this information indicates that impaired mitophagy is an important modulator of MAFLD progression to NASH and HCC.

## 4. Induction of Mitochondrial Biogenesis to Combat MAFLD

Current pharmacological treatment for MAFLD mainly repurpose drugs developed for type 2 diabetes and dyslipidemia. Insulin sensitizers such as pioglitazone activate PPARγ 1 and 2 in adipose tissue, thereby improving insulin sensitivity, and activate PPARα to reduce de novo lipogenesis [109]. Several studies show improvements in hepatic steatosis, hepatocyte ballooning and even fibrosis and weight gain [110,111].

GLP-1 receptor agonists such as liraglutide and semaglutide enhance glucose-dependent insulin secretion and delayed gastric emptying in addition to their weight loss induction. In MAFLD, they reduced steatosis and steatohepatitis and diminished triglyceride-based insulin resistance [112,113,114]. Farnesoid X receptor (FXR) agonists such as obeticholic acid modulate bile acid signaling while suppressing SREBP-1C, thus reducing lipogenesis. They showed improvements in steatosis and fibrosis despite frequently causing dose-dependent pruritus and atherogenic changes in LDL cholesterol levels [115]. PPAR agonists (both dual and pan agonists) increase fatty-acid oxidation, reduce adipogenesis and modulate anti-inflammatory pathways through PPARα/δ/γ activation, with some agents demonstrating histological improvements in NASH, although with heterogeneous efficiency across different compounds [116,117,118]. Sodium-Glucose cotransporter 2 (SGLT2) inhibitors reduce renal glucose reabsorption, promote glycosuria, and induce weight loss via reduction in visceral fat [119]. In MAFLD they have been shown to lower liver fat content while improving ALT and AST levels and inhibiting inflammatory cytokine release from adipocytes [120,121]. Antioxidant and anti-inflammatory agents, most notably vitamin E, have been demonstrated to improve steatohepatitis as well as AST and ALT levels in non-diabetic patients, although concerns have been raised about its long-term safety at higher doses [122].

Although several of these agents have shown improvements in MAFLD, their effects on mitochondrial quality control and oxidative metabolism remain largely unknown. As previously mentioned, only two compounds, those being resmetirom and semaglutide, have received FDA approval for MAFLD and NASH, highlighting the need for the discovery of new potential therapies. Due to increasing research on the role of mitochondrial dysfunction in MAFLD progression, targeting mitochondrial quality control pathways seems like a logical step forward in therapeutic development [4,14].

### 4.1. Lycium barbarum Polysaccharides (LBPs)

LBPs are bioactive polysaccharides extracted from *Lycium barbarum*. They have a long standing within traditional medicine and have been studied recently due to their antioxidant properties. Structurally, LBPs are heterogeneous polysaccharides composed of arabinose, galactose, glucose, mannose rhamnose and xylose, representing some of the most biologically active compounds present in the berry [123]. Their relevance to metabolic liver diseases has grown substantially as recent studies have demonstrated that LBPs exert beneficial effects on mitochondrial function, namely in the induction of mitochondrial biogenesis. In a MAFLD hepatocyte model, scientists showed that LBPs significantly increased mRNA expression of NRF-1 PGC1α and TFAM [124] (Table 1). Results also showed improved cell viability alongside a reduction in intracellular lipid accumulation and triglyceride content demonstrating an anti-steatotic effect [124].

Although this MAFLD-specific study did not identify the upstream signaling pathways responsible for this response, complementary evidence from other tissues helps shed light on the mechanisms behind LBP regulation of mitochondrial quality control. In a high-fat diet model of skeletal muscle atrophy, LBPs were shown to activate AMPK, which in turn promoted PINK1/Parkin-mediated mitophagy [125]. LBP beneficial effects were not observed in the presence of an AMPK inhibitor, further solidifying its role in the mechanism of modulation of mitochondrial function [125]. This study did not examine mitochondrial biogenesis directly; however, AMPK is a well-established activator of PGC1α, as was previously mentioned [126]. Activation of AMPK by LBPs in skeletal muscle cells suggests that this mechanism could be responsible for the induction of mitochondrial biogenesis in hepatocytes. LBP induction of mitophagy and biogenesis can act synergistically in order to maintain a healthier mitochondrial pool by enhancing clearance of damaged mitochondria while stimulating healthy mitochondrial synthesis [127].

Apart from biogenesis and mitophagy, LBPs have also been shown to influence mitochondrial dynamics. In the hyperglycemia-aggravated ischemic brain injury, there is an imbalance in mitochondrial dynamics characterized by an increase in DRP1 expression and decreased OPA1 expression, resulting in excessive mitochondrial fission and impaired fusion. LBP treatment reversed these changes, having significantly decreased DRP1 and increased OPA1 protein levels [128].

**Figure 3:**
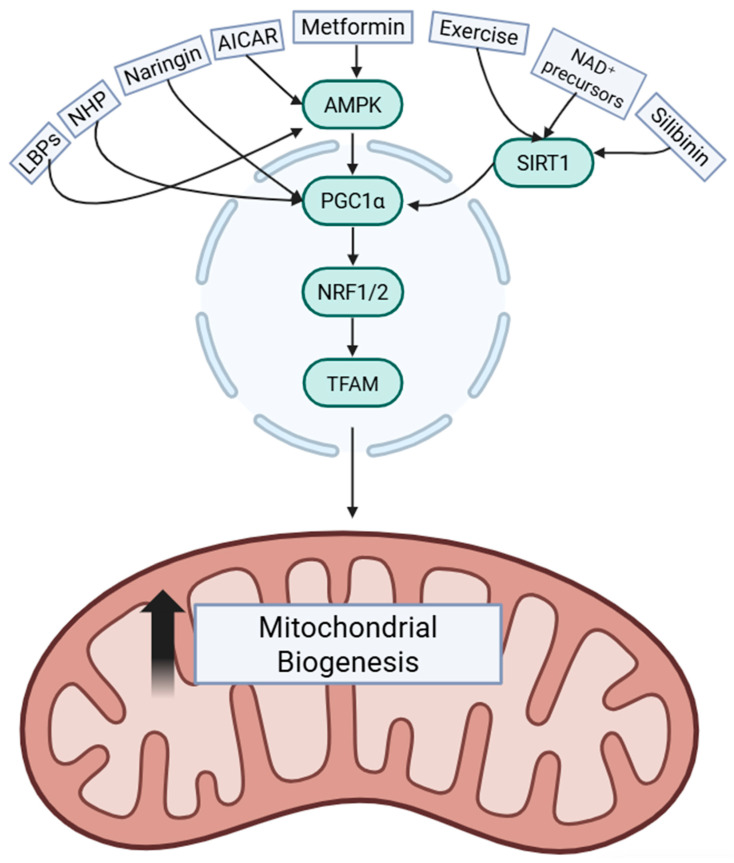
Schematic representation of the simplified mechanisms by which several strategies work to increase mitochondrial biogenesis. LBPs: *Lycium barbarum* polysaccharides; NHP: Neohesperidin; AICAR: 5-Aminoimidazole-4-carboxamide ribonucleotide; AMPK: Adenosine monophosphate-activated protein kinase; SIRT1: Sirtuin1; PGC1⍺: Peroxisome proliferator-activated receptor gamma coactivator 1-⍺; NRF: nuclear respiratory factor; TFAM: mitochondrial transcription factor A.

Although these findings were in extra-hepatic tissues, they reinforce the idea that LBPs act broadly on mitochondrial quality control, painting a picture of its possible mechanisms in the case of MAFLD treatment (Figure 3).

### 4.2. Neohesperidin

Neohesperidin (NHP) is a flavanone glycoside found in citrus fruits, with known metabolic benefits such as lowering blood glucose levels and having anti-obesity effects [129]. Its effect on hepatic steatosis and mitochondrial regulation has been recently clarified in a high-fat diet mouse model where NHP demonstrated hepatoprotective abilities. In high-fat diet-fed mice, NHP administration significantly lowered serum and hepatic ALT and AST levels. Additionally, it induced a reduction in hepatic triglyceride and cholesterol content as well as a decrease in lipid droplet accumulation [130]. These results demonstrate that NHP is able to suppress lipid overload in the liver under diet-induced stress.

NHP exerts its effects through enhancement of mitochondrial oxidative capacity. NHP significantly increased hepatic mtDNA content as well as ATP levels, indicating an increase in mitochondrial mass and capacity [130]. This increase in mitochondrial function was directly linked to the upregulation of mitochondrial biogenesis regulators. NHP treatment elevated hepatic mRNA levels of Nrf-1 and Tfam. NHP also increased both protein and mRNA expression of PGC1α in the liver of high-fat diet-fed mice (Table 1). In HepG2 cells, NHP restored *NRF-1* and *TFAM* gene expression, increased mitochondrial mass and enhanced ATP production and succinate dehydrogenase activity. All these effects were abolished by SR-18292, a selective inhibitor of PGC1α transcriptional activity, thus demonstrating that NHP-induced mitochondrial biogenesis is PGC1α-dependent [130].

Upstream, NHP was also shown to activate AMPK. In HFD-fed mice, NHP increased hepatic AMPK phosphorylation and in HepG2 cells, NHP stimulated AMPK activation in a dose-dependent manner. Palmitate suppressed AMPK phosphorylation, but NHP was able to reverse this suppression, indicating that it is able to restore AMPK activity under lipotoxic stress. Inhibition of AMPK with compound C prevented NHP-induced increase in PGC1α expression, confirming that AMPK activation is required for PGC1α upregulation by NHP [130].

NHP also upregulated fatty acid oxidation genes such as *Pparα*, *Acaa2*, *Cpt1*, *Pdk4* and *Acox1* while suppressing lipogenic genes such as *Srebf1*, *Fasn*, *Scd1* and *Ac1*. Beta-oxidation genes were comparatively more upregulated than lipogenic genes were downregulated, suggesting that an increase in Β-oxidation is the primary mechanism by which NHP aids in reduction in hepatic lipid accumulation [130]. This is consistent with the observed increase in mitochondrial biogenesis, as an enhancement in mitochondrial capacity leads to an increase in fatty acid catabolism (Figure 3).

### 4.3. Naringin

Much like neohesperidin, naringin is also a citrus-derived flavonone glycoside with well-documented antioxidant and lipid-lowering properties. Across multiple models of metabolic liver injury, naringin was able to reduce hepatic lipid accumulation while improving hepatocellular function and modulating mitochondrial homeostasis. In fructose-induced MAFLD, naringin decreased hepatic triglyceride and cholesterol content and reduced liver weight [131]. These effects were due to naringin’s suppression of endogenous triglyceride synthesis, reducing the expression of lipogenic proteins like SREBP-1C, ChREBP, FAS and ACC. Naringin also reduces the expression of Glycerol-3-phosphate acyltransferase 1 (GPAT-1) and DGAT2, reducing triglyceride synthesis transcriptionally. Naringin was also shown to modulate oxidative stress and inflammatory signaling in MAFLD, significantly reducing ROS levels while restoring NRF-2 and HO-1 expression [131].

In mitochondria, naringin has been shown to modulate mitochondrial dynamics and quality control in acetaminophen-induced hepatotoxicity. Although this is not an MAFLD model, the mitochondrial mechanisms here present should still be relevant. Naringin not only reduced but prevented acetaminophen-induced damage via modulation of mitochondrial dynamics and mitigation of ROS damage [132]. Naringin reversed the acetaminophen-induced decrease in mitochondrial fusion proteins MFN1 and OPA1, increasing their expression above control levels. It also reduced phosphorylation of DRP1 at Ser616, characteristic of mitochondrial fission [132]. Naringin was shown to activate AMPK and upregulated NRF-2. In the presence of an inhibitor of AMPK phosphorylation, NRF-2 activation was decreased, indicating that naringin regulates mitochondrial dynamics and antioxidant responses through AMPK [132].

When it comes to mitochondrial biogenesis, naringin alone is not able to significantly increase the expression of *SIRT1*, *PGC1α* or *TFAM*. However, when combined with caffeine, naringin contributed to the upregulation of all three genes [133] (Table 1). In HFD-fed rats this synergy was able to improve hepatic triglyceride content, despite treatment with both compounds separately not being enough. Histologically, this combination therapy was able to generate improvements in steatosis and hepatocyte ballooning while consequently decreasing inflammation [133]. Interestingly, the synergistic effect occurred despite that serum fatty acid levels remained unchanged across all treatment groups. These results indicate that improvements in triglyceride content were driven by intra-hepatic mechanisms rather than by changes in circulating lipids. The authors propose that the liver might sequester non-esterified fatty acids, storing them into triglycerides, and that this triglyceride accumulation might be a protective measure from lipotoxicity in the liver amidst the MAFLD to NASH transition [133].

### 4.4. AICAR

5-Aminoimidazole-4-carboxamide ribonucleotide (AICAR) is a purine nucleoside analog that is intracellularly converted to ZMP by phosphorylation, an AMP mimetic that activates AMPK by promoting phosphorylation of its catalytic α-subunit at Thr172 [134]. In human umbilical vein endothelial cells (HUVECs), a study showed that AICAR increased AMPK phosphorylation in parallel with a reduction in hyperglycemia-induced mitochondrial ROS [135]. In the same model, AMPK activation by AICAR upregulated PGC1α mRNA, increased NRF-1 and TFAM expression, and elevated mtDNA content and mitochondrial number (Table 1). Overexpression of dominant-negative AMPKα1 abolished these effects, confirming that AICAR’s mitochondrial effects are AMPK-dependent [135].

In C2C12 myotubes treated with AICAR, basal mitochondrial respiration was reduced but peak mitochondrial respiration and mitochondrial content were significantly increased, indicating a shift toward greater oxidative capacity under stimulated conditions [136]. Transcriptionally, AICAR increased NRF-1 expression at 6 and 12 h and Tfam at 6 h, without significant changes in Ppargc1a mRNA within the examined time frame, suggesting that AMPK activation can affect downstream targets even when PGC-1α transcriptional upregulation is not present [136]. Data from the previous two articles suggest AICAR activates AMPK, enhancing PGC-1α activity and its downstream transcriptional network, thus inducing mitochondrial biogenesis.

In MAFLD models, these mitochondrial effects translate into clear improvements in steatosis and metabolic homeostasis. In a study conducted by Zineldeen et al. high-fat diet (HFD)-fed rats developed obesity, insulin resistance, dyslipidemia, and MAFLD with macrovesicular steatosis, ballooning, inflammation, and elevated serum ALT/AST [137]. AICAR administration reduced body and liver weights, improved fasting glucose and insulin and HOMA-IR, corrected the lipid profile, lowered hepatic triglyceride content, and decreased MAFLD activity scores [137]. Electron microscopy revealed that HFD induced fragmented, swollen mitochondria with loss of cristae, whereas AICAR-treated livers displayed more numerous, tubular mitochondria with evidence of increased fusion. Morphometric analysis showed that AICAR increased the mitochondrial aspect ratio and form factor, indicating a shift away from fission and towards a more fused and interconnected mitochondrial network [137].

The same study demonstrated that AICAR downregulated hepatic Drp1 mRNA and upregulated SIRT2, a deacetylase known to restrain Drp1 activity and protect mitochondrial function. In palmitate-treated HepG2 cells, AICAR lowered Drp1 expression and increased SIRT2 even when AMPK was inhibited by Compound C, showing that AICAR modulates the SIRT2 in an AMPK-independent manner [137]. In parallel, AICAR increased CPT1A expression in an AMPK-dependent manner, enhancing mitochondrial β-oxidation, and upregulated CYP4F3 independently of AMPK, thereby promoting ω-oxidation as an alternative route for fatty acid disposal [137].

AICAR also modulates upstream inflammatory and hypoxic pathways that intersect with mitochondrial regulation in MAFLD. In the same HFD rat study, AICAR reduced serum HGF levels and attenuated hepatic NF-κB activation. This was shown by lower nuclear p65 and increased IκBα and decreased expression of the NF-κB target kinase SNARK, which negatively correlates with hepatic PGC1α expression [137]. AICAR lowered HIF-1α protein levels, restored SIRT2 expression, and increased PGC1α mRNA in the liver, indicating that it relieves HIF-1α-mediated repression of SIRT2-PGC-1α signaling. These changes were accompanied by higher hepatic NAD^+^ levels, reduced oxidative stress, and decreased pro-inflammatory cytokines (TNF-α, IL-6, and IL-1β) [137].

Another study showed that AICAR treatment in high-fat, high-fructose diet-fed mice reduced body weight gain, liver weight, hepatic triglyceride and total lipid content, and collagen deposition, while improving ALT/AST, insulin resistance, and oxidative stress markers [138]. In this model, AICAR increased hepatic p-AMPK and decreased p-mTOR levels, consistent with activation of the AMPK-mTOR axis, and upregulated FOXO3 mRNA and protein levels [138]. FOXO3 is a key regulator of antioxidant enzymes and mitochondrial homeostasis, and its upregulation by AICAR was associated with increased hepatic antioxidant capacity (SOD, CAT, GPx, and GSH) [138]. These findings align well with the endothelial study, where AMPK activation by AICAR induced PGC-1α, NRF-1, TFAM, MnSOD, and mitochondrial biogenesis, suggesting that in the liver, AICAR acts through the AMPK-PGC-1α-FOXO3 axis to enhance mitochondrial function and redox resilience (Figure 3).

### 4.5. Metformin

Metformin (1,1-dimethylbiguanide hydrochloride), a synthetic drug derived from galegine, has been used since the 1950s for the treatment of T2DM [139]. Metformin’s main action has been attributed to the inhibition of mitochondrial respiratory complex I, leading to an increase in the cellular AMP/ATP ratio and subsequent activation of AMP-activated protein kinase (AMPK) [140]. AMPK represses energy consuming anabolic processes such as gluconeogenesis and lipogenesis while promoting ATP-generating pathways, including fatty acid oxidation and mitochondrial biogenesis. Metformin also reduces reverse electron transport-driven ROS at complex I and protects against oxidative stress-induced cell death, demonstrating the importance of metformin’s mitochondrial mechanisms [141]. A key downstream consequence of AMPK activation by metformin is the engagement of the PGC1α-NRF-1-TFAM axis, the canonical transcriptional pathway for mitochondrial biogenesis [142].

In hepatocytes, metformin was shown to increase PGC1α mRNA and protein expression, a response that required AMPK activation and SIRT1, as inhibition of either pathway (compound C or SIRT1 siRNA) abolished the induction [143]. This effect was consistent across mouse and human primary hepatocytes and was also reproduced by adenoviral overexpression of constitutively active AMPK. Importantly, while metformin upregulated PGC1α and preserved its ability to induce mitochondrial genes, it selectively suppressed PGC1α-mediated induction of gluconeogenic genes such as *PEPCK* and *G6Pase* [143]. This selective modulation is further supported by metformin-induced PGC1α deacetylation, consistent with SIRT1 activation, and by the finding that metformin did not interfere with PGC1α-driven expression of mitochondrial proteins. Together, these results identify a mechanism in which metformin enhances hepatic mitochondrial biogenesis through AMPK-SIRT1-PGC-1α signaling while simultaneously preventing PGC-1α from activating gluconeogenic pathways [143] (Table 1).

In brown adipose tissue of mice fed a high-fructose diet, metformin increased the pAMPK/AMPK ratio and upregulated Pgc1α and Ucp1 expression, along with mitochondrial biogenesis markers Nrf1 and Tfam [144]. Metformin also raised the expression of Cpt1, enhancing mitochondrial fatty acid transport capacity, and induced Sirt1 and Fgf21, factors that further promote oxidative metabolism and thermogenesis [144]. Histologically, metformin increased BAT mass, restored the multilocular brown adipocyte morphology that was blunted by fructose, and induced browning features in subcutaneous white fat. These improvements in mitochondrial biogenesis markers accompanied by improved fatty acid oxidation are analogous to what is required to rescue impaired hepatic mitochondrial function in MAFLD, even though these particular data derive from adipose tissue.

In a β-glycerophosphate model of vascular calcification, metformin restored mitochondrial biogenesis that had been impaired by the osteogenic stimulus [145]. It increased mitochondrial DNA copy number, raised ATP levels, and upregulated PGC-1α, NRF-1, and TFAM expression in vascular smooth muscle cells (VSMCs) [145]. Immunofluorescence demonstrated that metformin promoted nuclear translocation of PGC1α, indicating enhanced transcriptional activity. All of these effects were abolished by the AMPK inhibitor compound C, showing that metformin’s biogenic action was AMPK-dependent in this context [145]. Importantly, restoration of mitochondrial biogenesis reduced oxidative stress, limited caspase-3/9 activation, and decreased apoptosis, which in turn attenuated the β-glycerophosphate-induced transition of VSMCs to an osteogenic phenotype. When mitochondrial biogenesis was blocked with zidovudine, mitochondrial membrane potential collapsed and apoptosis was exacerbated despite metformin, highlighting that the induction of mitochondrial biogenesis is functionally essential to the protective effect [145]. This VSMC study also revealed that metformin coordinates mitochondrial biogenesis with mitochondrial clearance through mitophagy. Metformin increased LC3-II and decreased p62, and autophagic flux assays with chloroquine confirmed the induction of mitophagy. Knockdown of Atg5 reduced mitophagy and concomitantly inhibited metformin-induced upregulation of PGC1α, NRF-1, TFAM, and mitochondrial DNA copy number, indicating that effective mitochondrial biogenesis required intact mitophagy [145]. In parallel, metformin suppressed β-glycerophosphate-induced expression of pyruvate dehydrogenase kinase 4 (PDK4), a mitochondrial enzyme whose upregulation increased ROS, decreased SOD activity, raised malondialdehyde levels, and promoted apoptosis. Inhibiting PDK4 with dichloroacetate reduced oxidative stress and apoptosis, and metformin mimicked this effect by lowering PDK4 via AMPK [145]. Together, these data show that metformin regulates mitochondrial homeostasis by coupling AMPK-driven biogenesis with mitophagy and by suppressing PDK4-mediated oxidative stress and apoptosis.

Functionally, metformin has reproducible benefits in MAFLD patients that are consistent with improved mitochondrial handling of lipids. A meta-analysis of randomized controlled trials showed that metformin in MAFLD reduced ALT and AST, lowered serum triglycerides and total cholesterol and improved insulin resistance while having little effect on BMI [146]. In animal models of diet-induced fatty liver, metformin reduces hepatic triglyceride content, improves histological steatosis, and normalizes ALT/AST. In a high-fat, high-fructose model, metformin activated AMPK in the liver and reduced mTOR signaling while also increasing FOXO3 expression [146]. FOXO3 is a known regulator of mitochondrial antioxidant enzymes and of mitochondrial homeostasis, and its upregulation by AMPK provides another route by which metformin can support mitochondrial integrity in hepatocytes [147].

It is also important to recognize that metformin’s mitochondrial effects in the liver are context- and dose-dependent. In Otsuka Long-Evans Tokushima Fatty (OLETF) rats with MAFLD and type 2 diabetes, metformin treatment alone improved HbA1c, reduced hepatic triglycerides, and lowered ALT, but did not significantly increase hepatic PGC-1α or cytochrome c protein content, nor did it enhance hepatic citrate synthase or β-hydroxyacyl-CoA dehydrogenase activity [148]. Exercise training, by contrast, robustly improved these mitochondrial indices, and combining metformin with exercise reduced some of the exercise-induced gains in hepatic β-hydroxyacyl-CoA dehydrogenase activity and complete palmitate oxidation [148]. These findings could mean that metformin is not a universal stimulator of hepatic mitochondrial biogenesis in every metabolic context and that its interaction with other mitochondrial stressors, such as exercise, can have potentially detrimental effects.

Additional work in metformin synergistic therapy has been performed in the context of MAFLD, showing improvements in metformin actions on disease parameters when combined with berberine. In HFD-fed mice, both agents individually improved hepatic steatosis, insulin resistance, and dyslipidemia, but their co-administration resulted in a stronger suppression of hepatic triglyceride and cholesterol accumulation, accompanied by a more pronounced reduction in MAFLD activity scores [149]. This combination elicited the greatest increase in hepatic AMPK phosphorylation, which corresponded to a robust inhibition of SREBP1 and its downstream lipogenic effector FASN, changes that were consistently observed in vivo and in OA/PA-loaded HepG2 cells [149]. Because AMPK directly suppresses SREBP1 maturation and nuclear translocation, the amplified AMPK response produced by dual therapy resulted in a deeper suppression of de novo lipogenesis and a more effective reduction in intracellular triglyceride content [149].

The requirement for AMPK in this synergistic response was confirmed by pharmacological inhibition. Compound C largely reversed the lipid-lowering effects of the metformin-berberine combination, restoring SREBP1 and FASN expression and re-accumulating triglycerides in hepatocytes [149]. These findings reinforce that metformin’s therapeutic value in MAFLD extends beyond its classical effects on gluconeogenesis. This indicates that AMPK activation, and the consequent suppression of SREBP1-driven lipogenesis, act as an important mechanism through which mitochondrial and metabolic homeostasis can be restored. While the previous study did not directly assess mitochondrial biogenesis, the enhanced AMPK signaling it documents is compatible with the broader literature showing AMPK-dependent induction of PGC-1α and mitochondrial biogenesis. This suggests that the lipid-lowering synergistic effects observed here likely coexist with the same upstream energy-sensing pathways that control mitochondrial adaptation in MAFLD.

### 4.6. Pseudolaric Acid B

Pseudolaric acid B (PAB) is a diterpene acid derived from red root bark. In high-fat diet (HFD) mice, PAB reduced body weight, improved serum triglycerides, total cholesterol, LDL cholesterol, and HDL cholesterol [150]. PAB also alleviated hepatic steatosis, reduced vacuolation, and lowered ALT/AST, indicating protection against hepatocellular injury. In hepatocytes exposed to free fatty acids, PAB consistently decreased intracellular triglyceride and total cholesterol content, reduced neutral lipid accumulation on Nile Red and Oil Red O staining, and lowered ALT/AST release without inducing apoptosis [150].

The study shows that PAB acts through direct activation of PPARα, a central regulator of mitochondrial fatty-acid oxidation and biogenesis. CETSA and DARTS assays confirmed physical binding of PAB to PPARα, while luciferase reporter assays demonstrated a near three-fold increase in PPARα transcriptional activity [150]. This activation upregulated downstream metabolic genes including *LPL*, *CPT-1α*, *CPT-1β*, *ATGL*, *HSL*, and *CD36*, and increased mitochondrial biogenesis markers PGC-1α and TFAM (Table 1). Functionally, PAB restored ATP levels, increased mtDNA content, and enhanced mitochondrial mass in both HepG2 cells and HFD mouse liver [150]. Importantly, the PPARα inhibitor MK886 reversed PAB’s effects on lipid accumulation, ATP, mtDNA, and downstream gene expression, confirming that PAB-induced improvements in mitochondrial function and lipid metabolism are PPARα-dependent [150].

### 4.7. SIRT1 Inducers

SIRT1 activation, regardless of the chemical class, consistently improves mitochondrial function and interrupts the metabolic and oxidative processes that drive MAFLD progression.

Aerobic exercise provides perhaps the most physiologically intuitive example of this axis. In the HFD-fed rat model, exercise robustly restored hepatic SIRT1 expression and corrected multiple hallmarks of mitochondrial dysfunction, including excessive Drp1-mediated fission, impaired antioxidant defenses, and reduced mtDNA content [151]. The study demonstrates that SIRT1-dependent deacetylation of Drp1 functions as an important regulatory mechanism. A high-fat diet increases Drp1 acetylation, leading to increased fission, whereas exercise reverses this acetylation state. Blocking SIRT1 with Tenovin-6 abolishes these benefits, reinstating Drp1 hyperacetylation, mitochondrial fragmentation, oxidative stress, and lipid accumulation [151]. In hepatocytes, pharmacological SIRT1 activation similarly rescues oleic acid-induced mitochondrial depolarization, ATP depletion, and loss of NRF-1/TFAM expression [151] (Table 1). One article utilized a zebrafish model to determine the effects of exercise on mitochondrial quality control. In diet-induced MAFLD, zebrafish exhibit fragmented mitochondria, reduced DRP1, OPA1, and MFN2, impaired oxidative phosphorylation, and suppression of the PINK1-PARKIN mitophagy pathway [152]. Exercise reversed these effects by activating the SIRT1/AMPK/PGC1α axis, increasing mitochondrial respiratory chain gene expression, and restoring mitochondrial size and morphology.

Natural products such as didymin, a citrus-derived flavonoid, extend this paradigm by combining transcriptional upregulation of SIRT1 with direct allosteric activation of the enzyme. Didymin binds to SIRT1 and enhances its deacetylase activity, leading to increased PGC1α signaling, elevated NRF-1 and TFAM expression in PA-treated hepatocytes [153]. In vivo, didymin improves MAFLD phenotypes in HFD-fed mice, reducing lipid deposition, normalizing serum lipids, and restoring mitochondrial ultrastructure. The study also highlights didymin’s ability to enhance lipophagy, suggesting that SIRT1-driven mitochondrial biogenesis and autophagic clearance of lipid droplets are tightly coordinated processes [153].

NAD^+^ precursors like nicotinamide riboside (NR) support SIRT1 activity by increasing cellular NAD^+^ levels upstream. Ethanol-induced liver injury, which shares some pathophysiological overlap with MAFLD, is characterized by depletion of hepatic NAD^+^, suppression of SIRT1 activity, and impaired PGC-1α deacetylation [154]. NR supplementation replenishes NAD^+^ pools, restores SIRT1 activity, and reactivates the PGC-1α-NRF-1-TFAM axis [154]. Importantly, SIRT1 knockdown abolishes NR’s protective effects, confirming that NAD^+^ repletion is not sufficient unless SIRT1 is present and functional.

A different but complementary strategy is exemplified by silibinin, the major bioactive component of silymarin. Silibinin reduces oxidative stress by inhibiting PARP-mediated NAD^+^ consumption, thereby preserving intracellular NAD^+^ and maintaining SIRT1 activity [155]. The study suggests that oxidative stress and NAD^+^ depletion form a self-reinforcing loop, and that silibinin breaks this loop by preventing excessive NAD^+^ loss.

Synthetic small molecules such as 4-butyl-polyhydroxybenzophenone derivatives have also been shown to upregulate SIRT1 and PGC-1α [156]. These compounds bind both proteins, enhance their interaction, and robustly induce mitochondrial biogenesis markers (SIRT1, PGC-1α, NRF-1, TFAM, COX1, and ND6) while reducing ROS, restoring ATP production, and increasing mitochondrial membrane potential [156]. In vivo, they reduce body weight gain, hepatic lipid accumulation, and serum injury markers in HFD-fed rats. The structural-activity relationship analysis, particularly the importance of ortho-hydroxyl groups for SIRT1/PGC-1α engagement, is a useful starting point for the design of next-generation mitochondrial therapeutics.

Mitochondria-targeted esculetin (Mito-Esc) has also been shown to enhance SIRT1-linked mitochondrial protection. By accumulating within mitochondria, Mito-Esc reduces mitochondrial superoxide, stabilizes membrane potential, and suppresses lipid accumulation in HepG2 cells [157]. In vivo, it improves steatosis, inflammation, and fibrosis in HFD-fed ApoE^−/−^ mice. Mito-Esc activates the AMPK-SIRT1 axis, and its effects are lost when AMPK or SIRT1 are silenced [157].

### 4.8. Phosphatidylethanolamine Metabolism Modulators

As previously mentioned, PE metabolism is a promising therapeutic axis in MAFLD and related hepatic disorders. One of the most direct therapeutic strategies is the restoration of mitochondrial PE levels. A study showed that chronic, but not acute, mitochondrial PE depletion led to impairments in mitochondrial biogenesis [74]. The same study demonstrated that lyso-PE supplementation normalizes mitochondrial PE content and rescues both morphology and respiratory function [74]. This suggests that targeted delivery of lyso-PE or enhancement of lyso-PE acylation pathways could ameliorate mitochondrial dysfunction in MAFLD. Because lyso-PE can be acylated within the ER and subsequently trafficked to mitochondria, this approach bypasses the need for PSD activity and directly replenishes the mitochondrial PE pool.

Additionally, Sam et al. showed that supplementation with ethanolamine (to stimulate the Kennedy pathway), lyso-PE (to feed acylation pathways), or even choline (to enhance PC synthesis) reduces the accumulation of misprocessed Psd1 and alleviates PE-deficiency stress [70]. These findings indicate that increasing ER-based phospholipid synthesis can indirectly support mitochondrial homeostasis by reducing the burden on mitochondrial import and processing pathways [70]. This suggests that therapies aimed at enhancing Kennedy pathway flux, such as ethanolamine supplementation or upregulation of PCYT2, may help stabilize mitochondrial PE levels and improve mitochondrial function in MAFLD (Table 1).

By targeting PE metabolism at multiple levels, it may be possible to restore mitochondrial biogenesis, normalize mitophagy, and interrupt the progression from steatosis to steatohepatitis and fibrosis.

## 5. Conclusions

In summary, accumulating evidence highlights that mitochondrial dysfunction is one of the main drivers of MAFLD progression, placing mitochondrial quality-control pathways as compelling therapeutic targets. Given its close association with metabolic dysfunction and multiple extrahepatic complications, effective strategies for prevention and treatment are needed across all stages of disease. Pre-clinical data have consistently demonstrated that targeting mitochondrial biogenesis pathways improves mitochondrial oxidative capacity and reduces lipid accumulation, two main cellular aggravators of MAFLD pathology. These findings indicate that mitochondria-oriented therapies are effective in experimental models of MAFLD. New therapies targeting mitochondria are actively being developed, with several different compounds having shown enough potential to progress to the clinical trial stage. Compounds like MitoQ, SS-31 and BGP-15 have shown promising results with minimal side effects in phase I clinical trials. To this day, no mitochondria-directed therapies for MAFLD have been approved for clinical use, highlighting the need for further research in the field.

Altogether, this article demonstrates the efficacy and importance of targeting mitochondrial quality control pathways, more specifically mitochondrial biogenesis, in the treatment of MAFLD. By compiling several strategies that induce mitochondrial biogenesis to treat MAFLD, we hope to shine a light on a growing body of promising research that could be used as a basis for the development of further therapies.

## Figures and Tables

**Table 1:** Mitochondrial targeted treatments of MAFLD.

Compound	Mechanism of Action	Experimental/Clinical Model	References
*Lycium barbarum* polysaccharides (LBPs)	Increase NRF-1, PGC1α, TFAM; reduce lipid accumulation; activate AMPK-dependent mitophagy (PINK1/Parkin); improve fusion–fission balance by lowering DRP1 and increasing OPA1.	MAFLD hepatocytes; HFD skeletal muscle atrophy; hyperglycemia-aggravated ischemic brain injury.	[123,124,125,126,127,128]
Neohesperidin (NHP)	Enhances mitochondrial biogenesis via AMPK-dependent PGC1α activation; increases mtDNA and ATP; boosts β-oxidation gene expression and suppresses lipogenesis.	HFD mice; HepG2 cells.	[129,130]
Naringin	Suppresses hepatic lipogenesis; reduces ROS and restores NRF-2/HO-1; improves mitochondrial dynamics; AMPK-dependent antioxidant signaling; supports biogenesis only when combined with caffeine.	HFD rats.	[131,132,133]
Caffeine	Enables naringin-driven increases in SIRT1, PGC1α, and TFAM, allowing mitochondrial biogenesis and improved hepatic lipid handling.	HFD rats.	[133]
AICAR	Activates AMPK to increase PGC1α, NRF-1, TFAM, mtDNA, and mitochondrial number; shifts mitochondria toward fusion; increases SIRT2 (AMPK-independent); enhances β- and ω-oxidation; reduces NF-κB, HIF-1α, oxidative stress, and inflammation.	HUVECs; C2C12 myotubes; HFD rats; HFHF mice.	[134,135,136,137,138]
Metformin	Activates AMPK via complex I inhibition; stimulates SIRT1-PGC1α-NRF-1-TFAM biogenesis; induces mitophagy; reduces ROS and PDK4; improves fatty acid oxidation; context-dependent effects on hepatic mitochondria.	Hepatocytes; BAT; VSMCs; MAFLD patients; HFD/HFHF rodents; OLETF rats.	[139,140,141,142,143,144,145,146,147,148,149]
Berberine	Strengthens metformin’s AMPK activation, producing stronger suppression of SREBP1/FASN and greater reduction in hepatic lipogenesis.	HFD mice; OA/PA-loaded HepG2 cells.	[149]
Pseudolaric acid B (PAB)	Directly activates PPARα, increasing fatty-acid oxidation genes and mitochondrial biogenesis markers (PGC1α, TFAM), increases ATP, mtDNA, and mitochondrial mass.	HFD mice; FFA-treated hepatocytes.	[150]
Exercise	Activates the SIRT1–AMPK–PGC-1α axis, increasing mitochondrial biogenesis through NRF-1 and TFAM; restores mitochondrial morphology by increasing OPA1 and MFN2 and normalizing DRP1; enhances oxidative phosphorylation and respiratory chain gene expression; increases β-oxidation; reduces ROS and inflammation.	High-fat diet rat model; high-fat diet zebrafish model.	[151,152]
Didymin	Direct SIRT1 activator that enhances PGC1α, NRF-1, TFAM and promotes lipophagy while improving mitochondrial structure.	PA-treated hepatocytes; HFD mice.	[153]
Nicotinamide riboside (NR)	Replenishes NAD^+^ to restore SIRT1 activity and reactivate the PGC1α-NRF-1-TFAM biogenesis pathway.	Ethanol-induced liver injury.	[154]
Silibinin	Preserves NAD^+^ by inhibiting PARP-mediated depletion, maintaining SIRT1 activity under oxidative stress.	Oxidative stress models.	[155]
4-butyl-polyhydroxybenzophenone derivatives	Enhance SIRT1–PGC1α interaction, increasing mitochondrial biogenesis markers and improving ATP, ROS balance, and membrane potential.	HFD rats.	[156]
Mito-Esculetin (Mito-Esc)	Mitochondria-targeted antioxidant that activates AMPK-SIRT1, reduces mitochondrial ROS, stabilizes membrane potential, and lowers lipid accumulation.	HepG2 cells; HFD ApoE^−/−^ mice.	[157]
Lyso-PE	Restores mitochondrial PE, thus rescuing morphology and respiration.	Yeast and mouse models of PE deficiency.	[70,71,72,73,74]
Ethanolamine/choline supplementation	Supports ER phospholipid synthesis and reduces Psd1 misprocessing.	Yeast and mouse models of PE deficiency.	[70,71,72,73,74]

## Data Availability

No new data were created or analyzed in this study. Data sharing is not applicable to this article.
